# Numerical analysis of intracellular amino acid profiles of breast cancer cells with K-Ras or PI3K mutation in response to kinase inhibitors

**DOI:** 10.1186/s12885-018-4972-7

**Published:** 2018-11-13

**Authors:** Jaesik Jeong, Kwangok Seo, Eung-Sam Kim

**Affiliations:** 1 Department of Statistics, Chonnam National University, 77 Yongbong-ro, Gwangju, 61186 Korea; 2 Department of Biological Sciences and Research Center of Ecomimetics, Chonnam National University, 77 Yongbong-ro, Gwangju, 61186 Korea

**Keywords:** Amino acid profile, Cancer metabolism, Cluster analysis, Kinase inhibitor, Principal component analysis

## Abstract

**Background:**

Various efforts to understand the relationship between biological information and disease have been done using many different types of highthroughput data such as genomics and metabolomics. However, information obtained from previous studies was not satisfactory, implying that new direction of studies is in need. Thus, we have tried profiling intracellular free amino acids in normal and cancerous cells to extract some information about such relationship by way of the change in IFAA levels in response to the treatment of three kinase inhibitors. We define two measures such as relative susceptibility (RS) and relative efficacy (RE) to numerically quantify susceptibility of cell line to treatment and efficacy of treatment on cell line, respectively.

**Methods:**

We applied principal component analysis (PCA) to the intracellular free amino acids (IFAAs) of isogenic breast cells with oncogenic mutation in K-Ras or PI3K genes to investigate the change in IFAA levels in response to the treatment of three kinase inhibitors. Two-dimensional plot, which was graphically represented by using the first two principal components (PCs), enabled us to evaluate the treatment efficacy in cancerous cells in terms of the quantitative distance of two IFAA profiles from cancerous and normal cells with the same treatment condition.

**Results:**

The biggest change in metabolic states in K-Ras mutant cell was caused by REGO for both treatment time (RS=2.31 (24 h) and 1.64 (48 h)). Regarding RE, REGO was the most effective on K-Ras/PI3K mutant cell line for treatment time 24h (RE=1.28) while PI3K inhibitor had good effect on K-Ras mutant cell line for 48h (RE=1.1).

**Conclusions:**

Numerical study on the link between amino acid profile and cancer has been done in two different dimensions. We then summarized such link in terms of two new metrics such as RS and RE, which we first define in this work. Although our study based on those metrics seems to work, we think that the usefulness of the metrics in cancer study of this kind need to be further investigated.

**Electronic supplementary material:**

The online version of this article (10.1186/s12885-018-4972-7) contains supplementary material, which is available to authorized users.

## Background

The reprogrammed metabolism observed in many types of cancer cells is known as an emerging hallmark of cancer. Altered metabolic pathways or oncometaboilites are to meet bioenergetics and biosynthetic demands of proliferating tumor cells [1–4]. Thus, cancer is recently viewed as a metabolic disease with altered metabolism, which drives many studies to analyze the change in the level of carbohydrate-, protein-, lipid-, or nucleic acid-based metabolites in cancer cells compared to normal cells [5]. Many genomic and proteomic studies have explored the change in the expression level of oncogenes as well as their mutation patterns in cancer cells. Although these oncogenes give rise to the change in metabolic pathways, few works on the experimental analysis of oncogene-specific profiles of intracellular metabolites, especially intracellular free amino acids (IFAAs) have been reported.

Among the numerous metabolites, amino acids have been considered as potential disease biomarker because they serve as not only building blocks for protein synthesis but also metabolic intermediators or regulators [6]. Thus, many studies have focused on revealing the interplay or link between the levels of amino acids and cancer. With plasma free amino acid (PFAA) profiles, some studies investigated the tumor-associated metabolism in cancer patients, being a potential biomarker of malignancy [7, 8]. Bi and Henry provided an excellent review on various methods which address the molecular and clinical associations between PFAA alterations and cancers [9]. However, it was also mentioned that the metabolic pathways leading to cancers is yet completely understood, i.e, still remains elusive.

The intracellular free amino acid (IFAA) profile can also indicate the altered metabolism due to the expression of oncogenic genes or treatment of small chemical inhibitors. We thus make use of the intracellular free amino acid (IFAA) profile as another route to the understanding of such interplay. We previously measured IFAA levels from the MCF-10A-derived cells with oncogenic mutations in K-Ras or PI3K genes [10]. The wild type (WT) MCF-10A cell was transformed to tumor-mimicking cells by the knock-in of mutant K-Ras and K-Ras/PI3K as oncogenes via the targeted knock-in method [11]. The IFAA profiles of the breast cancer-mimicking cells with or without the treatment of three types of inhibitors targeting multiple kinases, PI3K, and MEK, were also obtained to examine the inhibitor effect on the transformed cells.

The objectives of this work are to analyze the effect of oncogene expression on IFAA profiles and to evaluate the chemical intervention to the oncogene in terms of the similarity of IFAAs under treatment of three different kinase inhibitors. We investigate such link by way of two measures such as relative susceptibility (RS) and relative efficacy (RE). However, in high dimension, it is not easy to graphically represent the results. To avoid such difficulty, we employ principal component analysis (PCA), which summarize the high-dimensional information into low-dimensional one (especially 2D), i.e., dimension reduction. More specifically, we apply the PCA to the IFAA profiles and determine the effect of kinase inhibitors on the human breast cancer cells.

## Methods

### Biological experiment

**Fig. 1:**
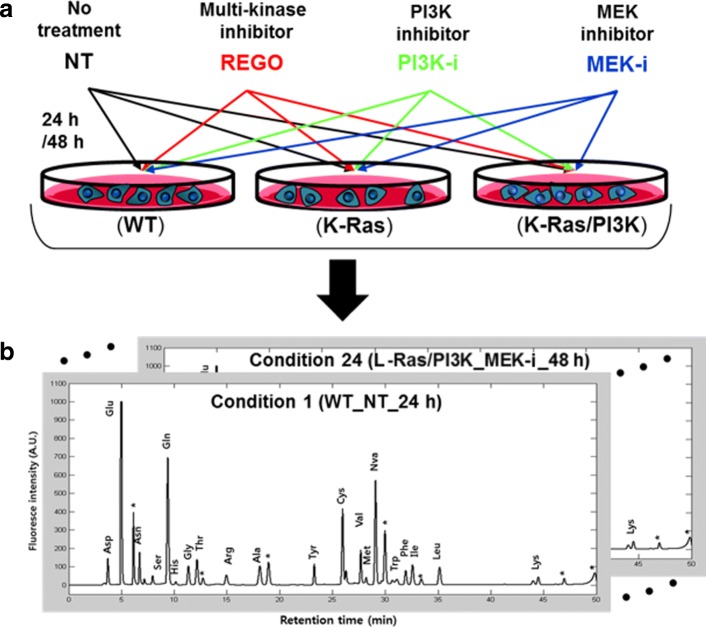
Overview of profiling free amino acids in cells. **a** Treatment of inhibitors to cells. The three types of isogenic MCF-10A cells including WT, K-Ras, and K-Ras/PI3K were treated for 24 or 48 h with three different kinase inhibitors: multiple kinase inhibitor (REGO), PI3K inhibitor (PI3K-i), and MEK inhibitor (MEK-i). Non-treated (NT) cells were used as controls. **b** Quantification of amino aicd levels. The IFAAs were extracted, chemically labeled, and separated to generate the amino acid profile in triplicate for 24 different conditions. The qunatified AA levels from each profile were represented into a 19-by-1 vector for the subsequent analysis. Note that unassigned peaks in an HPLC chromatogram were marked with *

The quantitative level of 19 IFAAs in the four types of MCF-10A cell lines was obtained after each cell line was treated with one of three kinds of kinase inhibitors as detailed in our previous work [10]. Briefly, the cells with knock-in mutation of K-Ras(G12V) and K-Ras(G12V)/PI3Ka(E545K) were named K-Ras and K-Ras/PI3K, respectively, compared to their wild type (WT) cells (Horizon Discovery, Cambridge, UK). These cells were cultured in DMEM:F12 (1:1) medium supplemented with 5% horse serum, 20 ng/ml epidermal growth factor (EGF), 10 *μ*g/ml insulin, 0.5 *μ*g/ml hydrocortisone, 0.1 *μ*g/ml cholera toxin, 100 U/ml penicillin, and 100 *μ*g/ml streptomycin. Cells were maintained at 37 ^∘^*C* with 5*%* CO _2_ in a humidified chamber. The three kinase inhibitors including REGO (i.e. Regorafenib or Stivarga ^TM^ approved by US FDA in 2012), PI3K-i (Bayer, Berlin, Germany) and MEK-i (Bayer) were treated to cells up to for 48 h in order to interfere multiple kinases, PI3K, and MEK, respectively (Fig. 1a). The chemical formula, structure, and dose of three inhibitors were listed in Additional file 1 (Table S1). Non-treated (NT) cells were used as controls. IFAAs were extracted from the pellet of 10^7^ cells according to the methanol extraction protocol [12]. Ortho-phthalaldehyde was used to label the extracted IFAAs with fluorophores excited and emitted at 345 nm and 455 nm, respectively. The fluorophore-labeled IFAAs were separated via reverse-phase HPLC (Applied Biosystems, Thermo Fisher, Waltham, USA) with Gemini-NX 5 *μ*m C18 100 Å column (Phenomenex, Torrance, USA) followed by quantification of peaks for assigned amino acids using standard curves (Fig. 1b). Experimental data are available in Additional file 2.

### Statistical measures

The following 19 amino acids among the 20 genetically-encoded amino acids were analyzed: alanine (Ala), arginine (Arg), asparagine (Asn), aspartic acid (Asp), cysteine (Cys); glutamine (Gln), glutamic acid (Glu), histidine (His), isoleucine (Ile), leucine (Leu); lysine (Lys), methionine (Met), phenylalanine (Phe), proline (Pro), serine (Ser), threonine (Thr), tryptophan (Trp), tyrosine (Tyr), and valine (Val). Since proline has no free amino group to be chemically labeled, it was excluded from our analysis. For the 24 conditions combining three cell types, four treatments, and two treatment times (24 h and 48 h), the triplicate IFAA profiles for each condition were represented as three 19-by-1 vectors with providing 72 vectors in total. Each condition was identified in the format of cell type$\underline {~}{inhibitor}\underline {~}$time (e.g. WT$\underline {~}{REGO}\underline {~}$24 h is for the condition of WT cell treated with REGO for 24 h). Note that the original 19 dimensional space is called original space and principal component space is called PC space.

We measured two different statistics which measure the degree of reaction of each cell line to the treatment and the efficacy of treatment on cancerous cell. Such statistics are represented as the Euclidean distance with reference to WT or NT, respectively. The distance between two IFAA profiles is calculated:

$$d(a,b)=\sqrt{ \sum\limits_{i=1}^{I}(a_{i} - b_{i})^{2}} $$

where both *a* and *b* are vector representing each condition and *I* is the dimension of the vector. For example, *a* and *b* could be the 2-dimensional PCs, each consisting of the first two principal components in PC space.

The susceptibility of cancerous cell (*Cell*) to each treatment (*Trt*) is defined:

$$s(Cell, Trt|WT)=d(Cell \underline{~} Trt,~ WT \underline{~} Trt). $$

As an example, the susceptibility of cancerous cell (*K*-*Ras*) to inhibitor (REGO) is

$$s(K\text{-}Ras, REGO|WT)=d(K\text{-}Ras \underline{~} REGO,~ WT \underline{~} REGO). $$

This means the susceptibility of K-Ras mutant cell to REGO with respect to WT, i.e., Euclidean distance between K-Ras mutant cell line and WT under the same experimental condition i.e., the same treatment and the same treatment time.

**Fig. 2:**
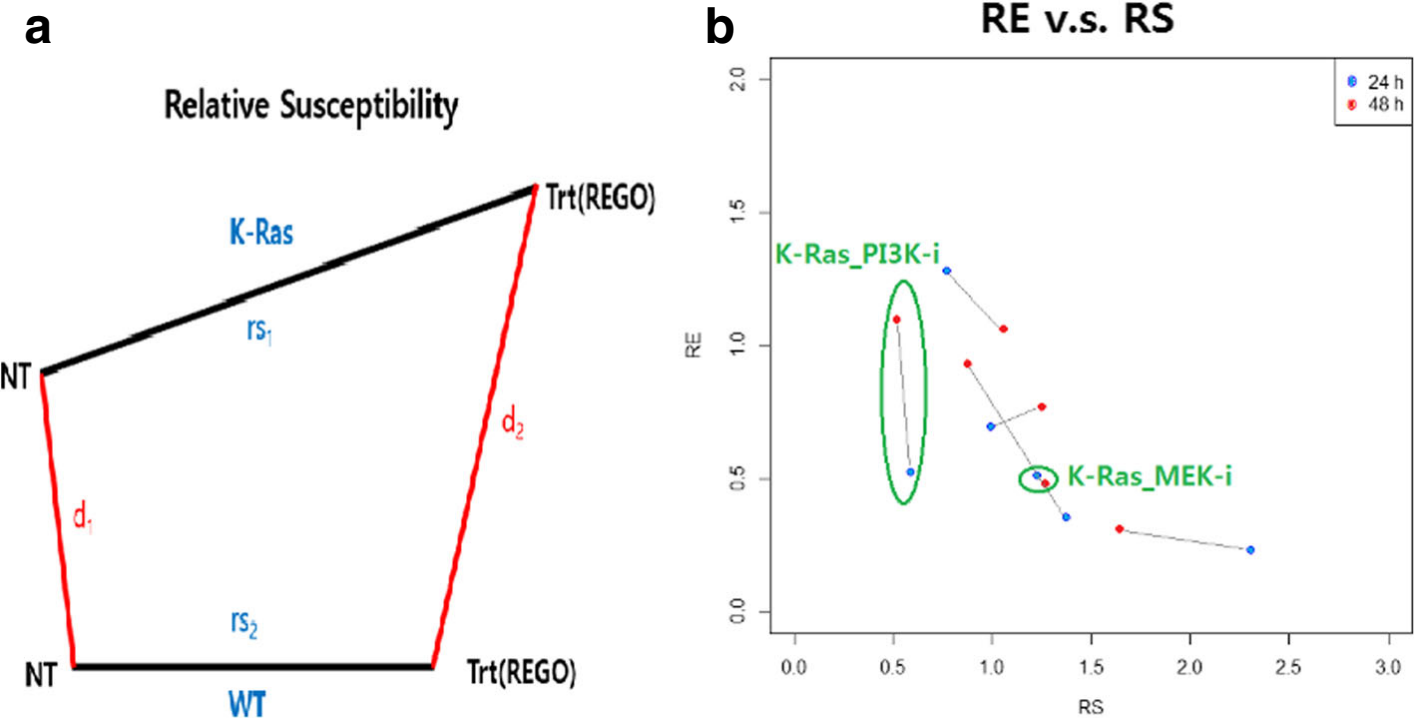
Two statistical measures. **a** Graphical representation of relative susceptibility and relative efficacy. **b** Plot of RE against RS for both incubation time (24h in blue and 48h in red). In the case that PI3K inhibitor is applied to K-Ras mutant cell, we observed big vertical change between two treatment time, more than twice increase in RE (the big circle in green). This means that we can expect some positive progress toward better situation after 48 h incubation time. In contrast, it is clear that there is little change in metabolic states 24 h after applying MEK inhibitor to K-Ras mutant cell line (the small circle in green)

With reference to WT, the relative susceptibility (RS) of cancerous cell to treatment (Trt) is defined:

1 $$ rs(Cell,~ Trt)=\frac{s(Cell,~Trt|NT)}{s(WT,~Trt|NT)}=\frac{d(Cell \underline{~} Trt,~ Cell \underline{~} NT)}{d (WT \underline{~} Trt,~ WT \underline{~} NT)}  $$

where *s* is the susceptibility of cell line to the same inhibitor with respect to NT. For example, the relative susceptibility of K-Ras to REGO is the ratio of two susceptibilities

2 $$ \begin{aligned} &rs(K\text{-}Ras,~ REGO)\\ &\quad=d(K\text{\,-\,}Ras \underline{~} REGO,~ K\text{\,-\,}Ras \underline{~} NT) / d(WT \underline{~} REGO,WT \underline{~} NT), \end{aligned}  $$

meaning the ratio of transition of K-Ras mutant cell to that of WT under the same condition with/without REGO treatment (Fig. 2a).

As another measure, we define the relative efficacy (RE) of treatment on cancerous cell:

$$re(Trt,~ Cell)=\frac{d(Cell \underline{~} NT,~ WT \underline{~} NT)}{d (Cell \underline{~} Trt,~ WT \underline{~} Trt)} \left(=\frac{d_{1}}{d_{2}} \right)$$

where the numerator and denominator are the distance between two cell lines before and after treatment, respectively (Fig. 2a). If the distance between two cell lines after treatment (*d*_2_) is relatively small compared to the distance before treatment (*d*_1_), it can be interpreted that the treatment has a positive effect on the mutant cell line. Equivalently, RE greater than 1 implies that inhibitor produces positive effect on the cancerous cell.

Note that the software R (available at https://www.r-project.org, ver. 3.2.3) was employed for all statistical analysis here.

## Results

### A global view: pattern in original space

We calculated two measures: relative susceptibility and relative efficacy. Those measures for the two treatment times are listed in Table 1 (graphically in Fig. 2b). For the treatment time of 24 h, the K-Ras mutant cell is the most susceptible to the REGO. That is, compared to other inhibitors, REGO perturbs the metabolic status most in K-Ras mutant cell line and PI3K inhibitor produces the least perturbation in the same cell. But, for K-Ras/PI3K mutant cell line, different pattern is observed. PI3K inhibitor produces the biggest move in the K-Ras/PI3K mutant cell line.

**Table 1:** Relative susceptibility (RS) and relative efficacy (RE)

Condition	RS(24)	RE(24)	RS(48)	RE(48)
(K-Ras, REGO)	2.31	0.23	1.64	0.31
(K-Ras, PI3K-i)	0.59	0.53	0.52	1.10
(K-Ras, MEK-i)	1.22	0.51	1.27	0.48
(K-Ras/PI3K, REGO)	0.77	1.28	1.05	1.06
(K-Ras/PI3K, PI3K-i)	1.38	0.35	0.88	0.93
(K-Ras/PI3K, MEK-i)	0.99	0.70	1.25	0.77

Two measures are calculated in the original space for two different times following treatment

Regarding relative efficacy, only one case (for the treatment time 24h) has meaningful value, i.e., greater than 1. More specifically, after REGO treatment, the cancerous cell (K-Ras/PI3K) moves closely toward the WT, showing an effective output. Similar patterns for all cases are observed for the treatment time of 48 h as well. However, in case of K-Ras cell treated with PI3K inhibitor, RE for that case changes from 0.53 to 1.1 as time goes, meaning that the K-Ras mutant cell gets closer to the WT after 24 h.

Ideally, we not only expect treatment to perturb the metabolic states as much as possible in cancerous cell, but also expect cancerous cell to move toward WT. In other words, it was expected that we observe some points with high RS as well as high RE (i.e., top right part of Fig. 2b). However, we observed some points with RE greater than 1 or RS greater than 1. Most interestingly, in the case that PI3K inhibitor is applied to K-Ras mutant cell, we observed big vertical change between two treatment time, more than twice increase in RE (the big circle in green in Fig. 2b). This means that we can expect some positive progress toward better situation after 48 h of treatment time. In contrast, it is clear that there is little change in metabolic states 24 h after applying MEK inhibitor to K-Ras mutant cell line (the small circle in green in Fig. 2b). More analysis results are given in Additional file 1 (Tables S2 and S3).

### PCA-based view: pattern in 2D-PC space

To investigate the validity of dimension reduction by PCA, we calculated the Euclidian distance between a given condition and the baseline condition (i.e., WT$\underline {~}\text {NT}\underline {~}$24 h) in two different spaces: Data space and PC space (Additional file 1: Table S4). As seen in the table, the two sets of distances from two different spaces are highly correlated (*R*^2^=0.99), implying the distance in PC space conserves the distance in the 19-dimensional original space very well. Clearly, the degree of change in IFAA profile can be appropriately translated and evaluated in the 2D-PC space. To graphically provide an overview of the global change of IFAA profiles for the 24 different conditions of oncogenic mutations and inhibitor treatments, we applied PCA to 24 averaged 19-by-1 vectors which were to be individually visualized into single points in two-dimensional principal component (2D-PC) space.

**Fig. 3:**
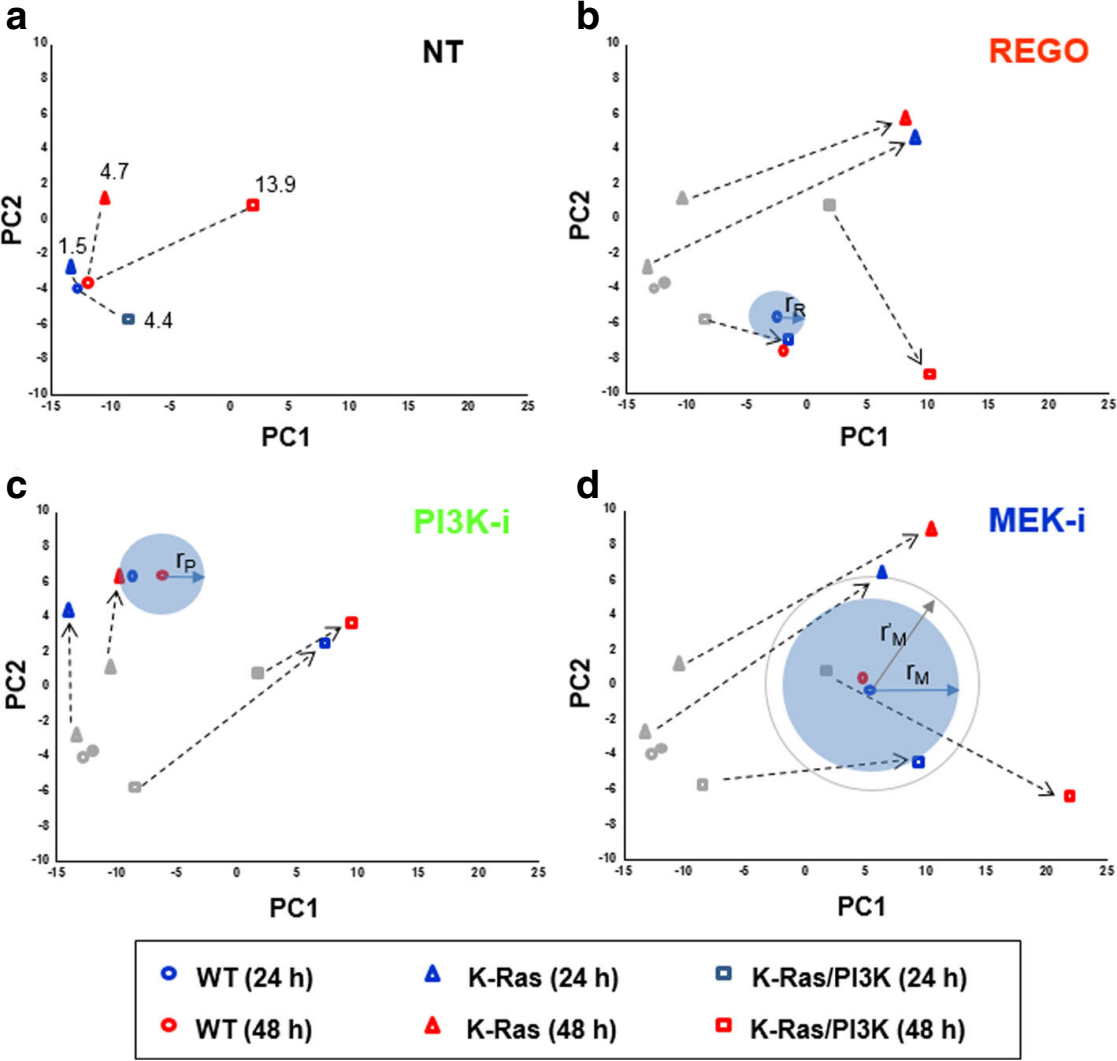
Global patterns of amino acids in response to oncogenic mutation and kinase inhibitors. The original PCA plot was divided into four graphs for each treatment. **a** NT (the numeric value is the distance between two IFAA profiles), **b** REGO, **c** PI3K-i and **d** MEK-i. For the treatment reference, the loci for the non-treated cells shown in **a** were depicted in gray in the other three plots. Each symbol (O: WT, △: K-Ras, and □: K-Ras/PI3K) colored in blue and red represents the IFAA of cells at 24 and 48 h, respectively. Dashed arrows were drawn to trace the temporal deviation of amino acid profiles in K-Ras and K-Ras/PI3K treated with each inhibitor. The three circles with radii, r_R_, r_P_, and r_M_ indicated a pair of cells (i.e. an inhibitor-treated WT and a mutant cell) with the most similar IFAA profile when treated REGO, PI3K-i, and MEK-i, respectively. *r*^′^_M_ is the distance for the second nearest pair of cells

Following the application of PCA, the variance explained by the first 9 PCs are summarized in Table 2. As seen in the table, since the first two principal components explain 85.6 percent of the total variance (PC1 and PC2 accounts for 65.8% and 19.8% of the total variance, respectively), we can comment on the relationship using the first two principal components without loss of generality (i.e., with probability of 0.86). The original PCA plot was separated into four plots, each representing the change in IFAA profiles under the four treatment types: No treatment (NT), REGO, PI3K-i, and MEK-i. In Fig. 3, relative transition of each inhibitor with respect to NT is represented. For example, Fig. 3b shows the transition made by inhibitor REGO in two different colors (24 h in blue and 48 h in red). As a reference, the results under no treatment are represented in grey. Two points obtained from a given treatment and NT under the same condition (the same mutant and the treatment) are connected by arrow in grey, implying susceptibility of each cancerous cell to each treatment. In practice, the length of the arrow implies the amount of relative effect of each inhibitor with respect to NT, i.e., distance between two situations. For all cases, the longest transition w.r.t NT is observed in Fig. 3d corresponding to MEK inhibitor. Clearly, we noticed that the treatment MEK-i perturb the metabolic status most significantly in all mutant cell lines. For reference, the results obtained by applying PCA to individual vectors first and then taking average of translated points are provided in Additional file 1 (Figure S2, Tables S5 and S6).

**Table 2:** PCA results on averaged vectors

Principal Component	Variance	Percentage of variance	Cumulative percentage
PC1	92.5	65.8	65.8
PC2	27.9	19.8	85.6
PC3	15.1	10.8	96.4
PC4	3.6	2.5	98.9
PC5	0.8	0.6	99.5
PC6	0.2	0.2	99.7
PC7	0.2	0.1	99.8
PC8	0.1	0.1	99.9
PC9	< 0.1	< 0.1	> 99.9

Variance explained by each principal components

For the non-treated cell, the distance of each cell line to WT is provided in Fig. 3a and Table 3. For 24 h culture time, the distance between K-Ras and WT (1.5) is about a third of that between K-Ras/PI3K and WT (4.4). As the culture time was increased, the IFAA profiles of the mutant cells were even more deviated from WT which showed a marginal deviation, suggesting that the higher number of oncogenic mutations and the longer culture time induce the more significant change in the IFAA profile.

**Table 3:** Euclidean distance in 2D-PC space

Tretment	Condition	24h	48h
NT	(K-Ras, WT)	1.5147	4.6979
NT	(K-Ras/PI3K, WT)	4.4387	13.8712
REGO	(K-Ras, WT)	15.3728	16.3767
REGO	(K-Ras/PI3K, WT)	1.7250	16.1222
PI3K-i	(K-Ras, WT)	5.8236	3.4196
PI3K-i	(K-Ras/PI3K, WT)	16.2723	15.8265
MEK-i	(K-Ras, WT)	6.9794	10.5205
MEK-i	(K-Ras/PI3K, WT)	5.7649	17.3562

The treatment of kinase inhibitors to cells resulted in further deviations of IFAA profiles, which was found to be dependent on the oncogenic mutation (Fig. 3b to d). The inhibitor-treated WTs (denoted by o) showed more distant IFAA profiles for the two incubation times in comparison to the non-treated WT. The treatment of kinase inhibitors to WT led IFAA profiles to be placed into the three different spots, indicating that each inhibitor have different actions in cell metabolism or signaling. In each treatment of inhibitors, the two IFAA profile of WT from 24 and 48 h treatments kept relatively short distances compared to the two IFAA profiles of mutant cells. That is, each cell line mainly react to an inhibitor within the 24 h after treatment, but not much after that. Notably, the treatment of REGO and PI3K-i to K-Ras and K-Ras/PI3K, respectively, shortened the distance of IFAA profiles for the two treatment times compared to their own non-treated profiles. In terms of deviation directions of IFAA profiles of mutant cells, REGO and MEK-i developed similar directions: right upward deviation in K-Ras and right or right downward in K-Ras/PI3K while PI3K-i induced upward and right upward in K-Ras and K-Ras/PI3K, respectively.

We also employed the clustering method to reveal the hierarchical clustering of IFAA profiles from 24 conditions. The non-standardized and standardized clustering could pair the two closest conditions with no quantitative distance (Additional file 1: Figure S3). However, the non-standardized clustering showed the closest pair for MEK-i treatment: WT$\underline {~}$MEK-i$\underline {~}$24 h and K-Ras$\underline {~}$MEK-i$\underline {~}$24 h (depicted with *r*^′^_M_ in Fig. 3d). The standardized clustering provided the same pair for three types of kinases as the PCA approach.

## Discussion

This work focused on the numerical analysis of IFAA profiles to assess the effect of chemical inhibitors on the metabolism of mutant cells, monitoring the chemical redirection of the metabolism of cancer cells into that of normal cells. Our evaluation criteria for the efficacy of the inhibitor is this similarity of metabolite profiles between the cancer and normal cells, not the significant difference in the number of dead cells when both cells treated with chemical inhibitors.

We analyzed the IFAA profile of cells with oncogenic aberration in K-Ras or PI3K genes compared to normal cells. The three different inhibitors were treated to normal and oncogenic cells to interfere the cellular metabolism, resulting in the change in IFAA profiles. Thus, these 24 profiles from the combination of three cell types, four treatment conditions, and two time points can represent the metabolic states of WT, K-Ras, and K-Ras/PI3K subjected to the treatment of REGO, PI3K-i, or MEK-i for 24 h and 48 h compared to the non-treated cells. Each profile contains quantitative levels of free 19 AAs out of 20 AAs building up proteins, which was represented into a single point in two different spaces: original (or 19-dimensional) and 2D-PC space. For the evaluation of the efficacy of the chemical inhibitors treated to normal and oncogenic cells, this work calculated the Euclidean distance of two points of interest. If the drug-perturbed IFAA profiles of normal and oncogenic cells are similar, the Euclidian distance of these two profiles with the same treatment time will be short. That is, the treatment condition in close vicinity to the perturbed normal cell can be determined as the most effective strategy to regulate the metabolism of oncogenic cells.

In the original space, we introduced two different measures such as relative susceptibility and relative efficacy, both of which are the ratio of two different Euclidean distances. However, in two-dimensional PC space, we used just Euclidean distance between two conditions. The main reason for the difference is that we lose some information when reducing dimension from 19 to 2. Furthermore, the amount of information lost depends on the position in the original space. In other words, the distance between two conditions in the original space is transformed to that in 2D space with some distortion depending on the position in the original space. Even though it is shown that the order of absolute distances is almost kept by way of pearson correlation coefficient, there is no guarantee that the ratio of distorted distances is kept. Therefore, we used different distance-based measures in each space.

In terms of the distance-based drug efficacy, the 24 h-long treatment of REGO to K-Ras/PI3K cells was found to be most effective in perturbing IFAA levels, finally inducing them to have a similar IFAA profile in comparison to WT cells with the same treatment condition. Its RE value and Euclidian distance (r _R_) in 2D-PC space were 1.28 and 1.7 (Tables 1 and 3, and Fig. 3b), respectively. Although the efficacy of MEK-i treated to K-Ras/PI3K cells (RE =0.70 to 0.77) was slightly higher than its treatment to K-Ras cells (RE = 0.51 to 0.48), both efficacy measures were much smaller than the REGO treatment of K-Ras/PI3K cells. It is known that the constitutively active form of K-Ras and PI3K mutant genes play key roles in development and progression of a wide range of human tumors. As a member of the Ras family, K-Ras stimulates multiple downstream signaling pathways such as PI3K-Akt and Raf-MEK-ERK [13]. Since most effectors of K-Ras are kinase porteins that promote cell proliferation, growth and cell survival [14], the action of mutant K-Ras and PI3K genes might synergistically give the mutant cell more cancer-like features in our in vitro culture [10] showing the colony-formed cell division compared to the typical (i.e. monolayer-formed) cell growth of WT. Thus, it can be speculated that a multi-kinase inhibitor, REGO suppresses the action of some kinases in the K-RAS or PI3K-downstreamed signaling cascades although its target is not a specific kinase such as PI3K and MEK. However, the treatment of REGO to K-Ras mutant cells could not bring the equivalent efficacy (RE =0.23 to 0.31, r _R_ =15.4) as did its treatment to K-Ras/PI3K cells. Further exploration on how the mutant genes individually or synergistically contribute to metabolic reprogramming through Ras-mediated pathways will be required to understand this discrepancy.

The nearest profile pair under PI3K-i was K-Ras and WT (r _P_ = 3.4 in Fig. 3c and Table 3) when both treated for 48 h while its RE value was 1.10. The inhibition of MEK with the treatment of MEK-i was found to be unsuccessful in inducing them to have the most similar IFAA profiles. We had expected that MEK-i also could allow remarkable efficacy on the assumption that both PI3K-Akt and Raf-MEK-ERK pathways be almost equivalent under the mutant K-Ras. The efficacy of the 24 h-long treatment of PI3K-i (RE =0.53, r _P_ = 5.8) was smaller than its 48 h treatment. These results suggest that the 48 h-long treatment of PI3K-i to K-Ras mutant cells is the most effective inhibitor compared to other treatment conditions. Thus, the treatment time should be also optimized to achieve better efficacy for a given set of an inhibitor and mutant cells.

## Conclusion

A number of works have made efforts to profile metabolites of cells to assess their metabolic states, especially to define caner-associated metabolites as cancer biomarkers. Our work allowed taking a step forward in understanding the change of intercellular metabolite due to oncogene expressions, which will offer another insight into tumorigenesis when combined with tumor-associated genomic or proteomic alterations in cells. Although the IFAA profile is a part of the whole list of metabolites, it can be proposed that the change pattern of the IFAA profile be used as an indicator of the drug efficacy. When it is assumed that an inhibitor affects both cancer and its adjacent normal cells in the body for the cancer control, one of the therapeutic aims is to lead the metabolic states of the cancerous cell to be close to those of the normal cell under the same inhibitor. Since cancer cells reprogram their metabolism to be deviated from the normal cell, the similar metabolic states of cancer and normal cells may indicate that cancer cells are exposed to a fatal situation due to the drug interference. In this respect, we defined two different measures and partly observed weak, yet positive signal in the cancerous cell line after treatment. However, our proposal for IFAA-based drug efficacy requires further preclinical or experimental validation. Especially, if there are multiple treatment conditions of cancer cells which are almost the same distance away from the normal cell, the issue of which treatment is to be selected should be resolved later.

## Additional files

Additional file 1 This file includes additional results such as figures and tables. (PDF 421 kb)

Additional file 2 All data set used in the paper are included in this file. (XLSX 36 kb)

## Abbreviations

IFAA: Intracellular free amino acids
PCA: Principal component analysis
PFAA: Plasma free amino acids
RE: Relative efficacy
REGO: Regorafenib
RS: Relative susceptibility
WT: Wild type
