# Supplementary material for: Numerical analysis of intracellular amino acid profiles of breast cancer cells with K-Ras or PI3K mutation in response to kinase inhibitors
Source: BMC Cancer. 2018 Nov 13;18:1109. doi: 10.1186/s12885-018-4972-7 (PMC6234589; doi:10.1186/s12885-018-4972-7)
Supplement: Supplementary file 1 — This file includes additional results such as figures and tables. (PDF 421 kb) [file 12885_2018_4972_MOESM1_ESM.pdf]

Supplementary Materials: Numerical analysis of intracellular amino acid profiles of breast cancer cells with K-Ras or PI3K mutation in response to kinase inhibitors

Jaesik Jeong[1], Kwangok Seo[1] and Eung-Sam Kim[2*]

[1] Department of Statistics, Chonnam National University, Gwangju, 61186 Korea
[2] Department of Biological Sciences, Chonnam National University, Gwangju, 61186 Korea

Chemical information of three inhibitors

Four different chemical information for three inhibitors are provided below:

Table 1: Chemical information of three inhibitors

	REGO***	PI3K-i	MEK-i
Chemical formula	$C_{22}H_{16}CIF_4N_3O_3$	$C_{22}H_{26}N_6O_3$	$C_{17}H_{17}F_2IN_2O_4$
Structure	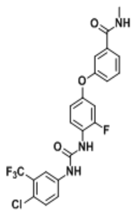	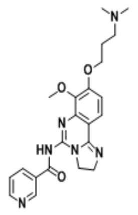	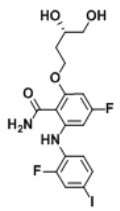
Final concentration(μM) *	10	5.0	3.0
Solvent for 10 mM ** stock solution	Anhydrous DMSO	DMSO supplemented with 10mM trifluoroacetic	Anhydrous DMSO

* The final concentration represents IC_{50} value of each inhibitor treated to K-Ras cells. All IC_{50} values were determined according to the manufacturer's instruction(Promega).
** All stock solutions were stored in the dark at room temperature.
*** REGO: its full name is regorafenib as shown at https://pubchem.ncbi.nlm.nih.gov/compound/Regorafenib.

Relative susceptibility and relative efficacy

We calculated two measures for two different treatment time: relative susceptibility and relative efficacy. The following tables (Tables S2 and S3) include those measures. d_1 and d_2 are defined in Figure 2A in main text.

Comparison of variances of two PCA applications

To investigate the validity of dimension reduction by PCA, we calculate the Euclidian distance between a given condition and WT_NT_24 h in two different space: Data space and PC space (Table S4).

Table 2: Treatment time 24h: Relative susceptibility and relative efficacy

Condition	RS	RE	d_1	d_2
(K-Ras, REGO)	2.31	0.23	3.63	15.63
(K-Ras, PI3K-i)	0.59	0.53	3.63	6.91
(K-Ras, MEK-i)	1.22	0.51	3.63	7.10
(K-Ras/PI3K, REGO)	0.77	1.28	5.80	4.53
(K-Ras/PI3K, PI3K-i)	1.38	0.35	5.80	16.35
(K-Ras/PI3K, MEK-i)	0.99	0.70	5.80	8.35

Table 3: Treatment time 48h: Relative susceptibility and relative efficacy

Condition	RS	RE	d_1	d_2
(K-Ras, REGO)	1.64	0.31	5.25	16.88
(K-Ras, PI3K-i)	0.52	1.10	5.25	4.78
(K-Ras, MEK-i)	1.27	0.48	5.25	10.86
(K-Ras/PI3K, REGO)	1.05	1.06	15.18	14.28
(K-Ras/PI3K, PI3K-i)	0.88	0.93	15.18	16.27
(K-Ras/PI3K, MEK-i)	1.25	0.77	15.18	19.66

As seen in the table, the two sets of distances from two different space are highly correlated ($R^2 = 0.99$), implying the distance in PC space conserves the distance in the 19-dimensional original space. That is, it is clear that the degree of change in IFAA profile can be appropriately translated and evaluated in the 2D-PC space.

Intracellular free amino acids (IFAA) profiles were obtained from 24 different experimental conditions. For each condition, three IFAA profiling experiments were independently repeated to confirm the reproducibility. The principal component analysis (PCA) was applied to the raw data (i.e. 72 19-by-1 vectors in total), which is called Individual. Also, we applied the PCA to the 24 averaged data, which is called Average. Depending on the data set to which the PCA is applied, it is named: Individual and Average. The following tables provide the proportion of variance explained by each principal component. Corresponding numerical numbers are given in Tables S5 and S6 below. The two tables include the first nine components without listing the other ten components whose contributions to total variance were negligible (i.e. less than 0.1 in variance).

Effect of calculation order on PCA plots

Due to the difficulty of graphical representation of high-dimensional data, we applied PCA to the raw data, and then focused on the first two principal components, i.e., two dimensional PC space. Since two events such as averaging and PCA are applied to our data, there might be the order effect. The meaning of the order effect is whether the fact that which event is first applied affects the results or not. To see if there is the order effect, we compare two different scenarios: (i) application of average followed by PCA, (ii) application of PCA followed by average. Two results are represented in Figure S1. When the total 72 vectors were input to PCA, the centroid of the three points for a given condition was almost identical to the point after applying PCA to the averaged vector, showing no significant difference between the two approaches to obtain the PCA plot (Fig. S1). Thus we here make use of the transformed point of averaged vectors by PCA. Such 24 points are given in Figure S2. Among them, six points corresponding to each treatment are highlighted in different figures: Figure S2 (A) to (D).

Table 4: Euclidian distances between WT_NT_24h and each condition in 2-dimensional PC and 19-dimensional spaces

Conditions	Distances in 2-dimensional PC space	Distances in 19-dimensional space
WT_NT_24h	0.00	0.00
K-Ras_NT_24h	1.51	3.63
K-Ras/PI3K_NT_24h	4.44	5.80
WT_REGO_24h	10.12	10.64
K-Ras_REGO_24h	23.25	23.84
K-Ras/PI3K_REGO_24h	11.31	11.50
WT_PI3K-i_24h	11.22	13.16
K-Ras_PI3K-i_24h	8.55	10.28
K-Ras/PI3K_PI3K-i_24h	20.81	22.08
WT_MEK-i_24h	17.87	17.92
K-Ras_MEK-i_24h	21.72	21.76
K-Ras/PI3K_MEK-i_24h	21.69	22.32
WT_NT_48h	0.85	3.33
K-Ras_NT_48h	5.43	6.81
K-Ras/PI3K_NT_48h	14.62	15.35
WT_REGO_48h	11.23	13.41
K-Ras_REGO_48h	22.88	24.01
K-Ras/PI3K_REGO_48h	23.27	23.46
WT_PI3K-i_48h	12.18	13.15
K-Ras_PI3K-i_48h	10.69	11.38
K-Ras/PI3K_PI3K-i_48h	23.48	24.67
WT_MEK-i_48h	18.39	18.47
K-Ras_MEK-i_48h	26.50	26.62
K-Ras/PI3K_MEK-i_48h	34.37	35.46

Representation of hierarchical clustering with heatmaps

A two-dimensional heatmap (or heat map) displays the results of a cluster analysis by permuting the rows and columns of a matrix-formed data to place similar values near each other with representing the numerical values into colors. Generally, rows or columns with large values dominantly influence the cluster analysis in the heatmap in comparison with other rows or columns containing small values. As seen in Fig. S3 (A), most columns of the heatmap have similar colors except the last three columns with relatively large numbers. The clustering result of the standardized data can be graphically represented into a heatmap as shown in Fig. S3 (B).

Table 5: Variance for each principal component of PCA applied to 24 averaged vectors

Principal components	Variance	Percentage of variance (%)	Cumulative percentage (%)
PC1	92.5	65.8	65.8
PC2	27.9	19.8	85.6
PC3	15.1	10.8	96.4
PC4	3.6	2.5	98.9
PC5	0.8	0.6	99.5
PC6	0.2	0.2	99.7
PC7	0.2	0.1	99.8
PC8	0.1	0.1	99.9
PC9	<0.1	<0.1	>99.9

Table 6: Variance for each principal component of PCA applied to72 raw vectors and subsequently averaged in PC space

Principal components	Variance	Percentage of variance (%)	Cumulative percentage (%)
PC1	100.7	59.3	59.3
PC2	35.4	20.9	80.3
PC3	17.9	10.5	90.7
PC4	12.5	7.3	98.1
PC5	1.5	0.9	99.0
PC6	0.9	0.5	99.5
PC7	0.4	0.2	99.7
PC8	0.3	0.2	99.9
PC9	0.1	<0.1	>99.9

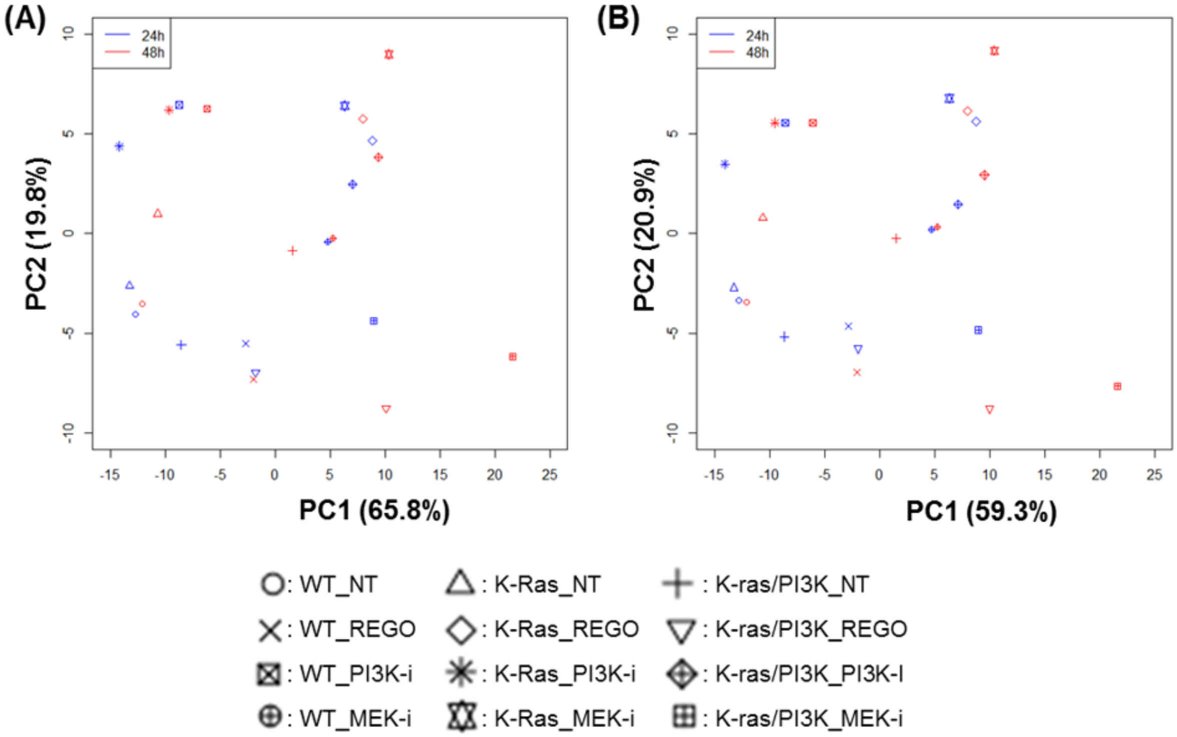

Figure 1: Comparison of PCA plots. (A) PCA plot for 24 averaged vectors. (B) PCA plot for 72 raw vectors which were averaged into 24 loci in the PC space (i.e. one loci of the PCA plot is the centroid of the three points to which three raw vectors corresponding to a treatment condition are projected).

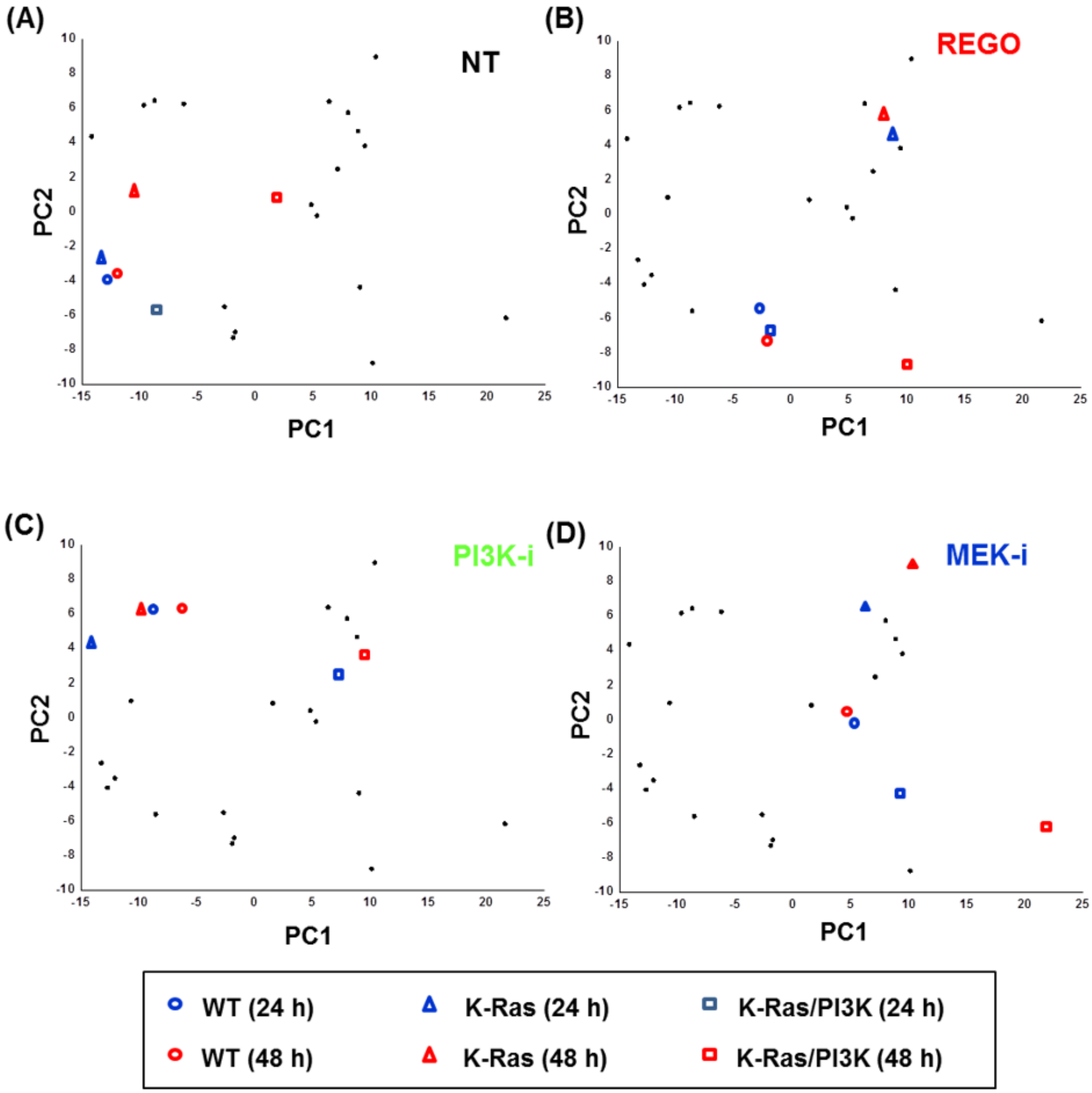

Figure 2: Original PCA plots with 24 loci. (A) The three types of non-treated (NT) cells are highlighted. The three types of cells treated with REGO, PI3K-im and MEK-I are highlighted in (B), (C) and (D), respectively.

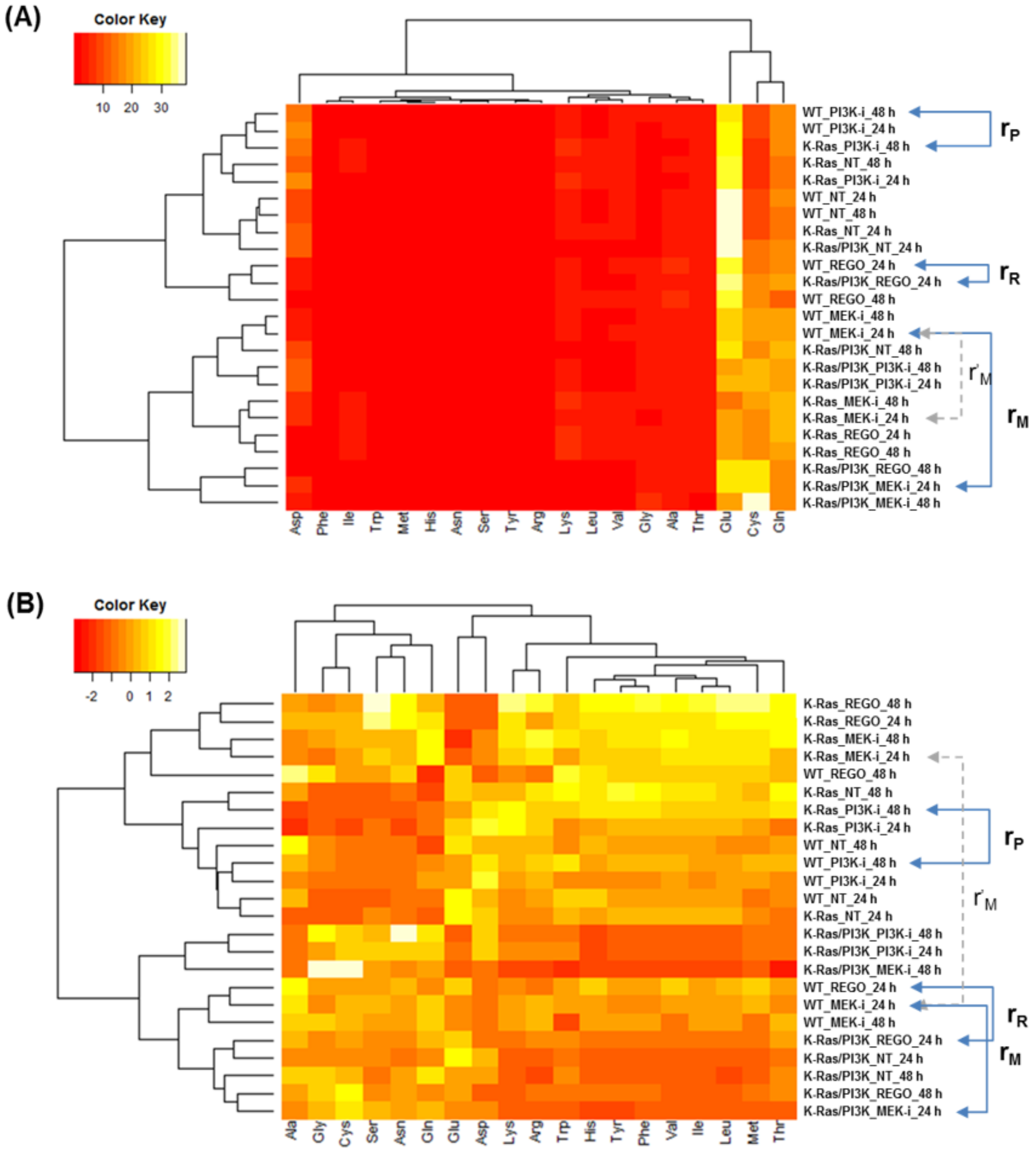

Figure 3: Heatmaps to represent the result of clustered groups of conditions. (A) Non-standardized clustering. (B) Standardized clustering.
